# Digit Ratio Predicts Sense of Direction in Women

**DOI:** 10.1371/journal.pone.0032816

**Published:** 2012-02-29

**Authors:** Xiaoqian J. Chai, Lucia F. Jacobs

**Affiliations:** 1 Department of Brain and Cognitive Sciences, Massachusetts Institute of Technology, Cambridge, Massachusetts, United States of America; 2 Department of Psychology, University of California, Berkeley, California, United States of America; The University of Western Australia, Australia

## Abstract

The relative length of the second-to-fourth digits (2D:4D) has been linked with prenatal androgen in humans. The 2D:4D is sexually dimorphic, with lower values in males than females, and appears to correlate with diverse measures of behavior. However, the relationship between digit ratio and cognition, and spatial cognition in particular, has produced mixed results. In the present study, we hypothesized that spatial tasks separating cue conditions that either favored female or male strategies would examine this structure-function correlation with greater precision. Previous work suggests that males are better in the use of directional cues than females. In the present study, participants learned a target location in a virtual landscape environment, in conditions that contained either all directional (i.e., distant or compass bearing) cues, or all positional (i.e., local, small objects) cues. After a short delay, participants navigated back to the target location from a novel starting location. Males had higher accuracy in initial search direction than females in environments with all directional cues. Lower digit ratio was correlated with higher accuracy of initial search direction in females in environments with all directional cues. Mental rotation scores did not correlate with digit ratio in either males or females. These results demonstrate for the first time that a sex difference in the use of directional cues, i.e., the sense of direction, is associated with more male-like digit ratio.

## Introduction

The ratio of the second to the fourth finger length on the right hand (2D:4D, or digit ratio) is putatively a marker of the organizational effect of prenatal testosterone in humans ([1], see [2], [3], [4], [5] but see [6], [7]). It is sexually dimorphic, with women having higher ratios than men on average [8], [9], [10]. It has been hypothesized that 2D:4D reflects prenatal androgen levels and the individual's sensitivity to androgens [2]. There is indirect evidence that supports such hypothesis in humans. Higher (more feminized) 2D:4D ratios were reported in females with androgen insensitivity syndrome [6]. Individuals with congenital adrenal hyperplasia (CAH), a condition associated with high levels of prenatal androgens, have been found to have smaller 2D:4D ratio [11], [12], [13], [14]. Sexual dimorphism in digit ratio has also been found in a number of non-human species, including rodents [15] and anthropoid primates [16]. A study that directly manipulated hormone level in pregnant rats showed that elevated level of maternal testosterone resulted in lower 2D:4D ratios in offsprings [17]. The relative level of prenatal testosterone and oestrogen signaling during a narrow window of fetal development has been recently shown to have a causal effect on 2D:4D in mice ([18], see [19]).

Many spatial abilities are sexually dimorphic and appear to be influenced by prenatal testosterone [20]. Therefore one might expect digit ratio to correlate with spatial ability. However, previous research on the relationship between spatial ability and digit ratio has produced mixed results [21], [22], [23], [24]. Some found no significant correlations between digit ratio and spatial ability [10], [25], [26], others have reported both negative [23], [24] and positive correlations [10] in males or females. A recent meta-analysis by Puts, et al. (2008) [27] analyzed the effect size (correlation coefficients between 2D:4D and spatial ability), and found the effect size across studies to be negligible, for either males or females. However, the majority of the studies published so far, including the ones reviewed by Puts et al. (2008) assessed spatial ability using only two-dimensional tasks, such as the mental rotation test (MRT). Yet it is unclear how such tasks relate to performance in spatial navigation [28]. To our knowledge, only two studies have examined the relationship of digit ratio and navigation with a three-dimensional (3D) spatial task. Both studies used a maze-learning task adapted from the rodent Morris water maze [26], [29]. Neither study found the expected relationship between digit ratio and spatial abilities. The Morris water maze requires participants to navigate to a hidden platform within the test arena using external distal cues. Males typically need less time (latency to target) before learning the target location in virtual simulations of this type of maze [28], [30]. Because of the robust sex difference in Morris water maze, which favors males, one might expect a lower digit ratio (i.e., more masculine) to be associated with shorter latencies. Yet Csatho et al. (2003) [29] reported that lower digit ratio (i.e., more masculine) was associated with a longer search latency in females. The authors also reported that lower digit ratio was associated with better post-test navigational cue identification. This result was also not expected since females often outperform males in object location recall [31], [32], [33], [34]. A higher ratio (more feminine) might be expected to correlate with better cue identification. This important study has therefore raised many open questions.

A possible reason for this conflicting finding on the relationship between digit ratio and navigation abilities in the Csatho et al. (2003) study is the nature of the tasks being used. Spatial navigation most likely recruits multiple cognitive abilities, and the recruitment of the specific ability may vary between the sexes, with females and males relying on different sets of cues to orient. In rodents, females are notably sensitive to the unique features of discrete objects whereas males are sensitive to extra-maze cues such as the geometry of the enclosure [35], [36], [37], [38], [39], [40]. Similar results have been shown in humans [41], [42]. Males relied more on geometric information than females [41], whereas females are often more sensitive to the switching of local object location [31], [43]. This raises the possibility that the relationship between digit ratio and navigation performance is particularly sensitive to the types of cues that are available in the environment. For example, cues that are preferentially used by males, such as distant cues and geometrical shape of the space, should be more closely correlated with lower digit ratio.

In the parallel map model of the cognitive map [44], the map is created by integrating information from two distinct functional cue classes: directional and positional cues. Directional cues provide primarily directional (compass) information. Distal landmarks, for example, are too far away to provide accurate positional information but can nevertheless give a directional bearing. Similarly, extended cues such as gradients (odor, light or terrain slant), or geometric cues primarily provide directional information. In contrast, positional cues are discrete and local objects, which provide relatively precise positional information within a local cue array. The model predicts a male advantage in environments that are rich in directional cues and female advantage in environments that are rich in positional cues. Evidence supporting this prediction was reported from tasks involving manipulations of directional and positional cues in the stimuli [42], [45].

Given the possibility that the functional class (whether primarily directional or positional) of cues in a spatial environment might affect the performance of males and females in different ways, one interpretation of the anomalous results in the Csatho et al. (2003) [29] study, is that the physical layout of the task could have prevented the use of the male-preferred directional cues. The maze used in the Csatho et al. (2003) study was a circular arena containing intra-maze object cues (i.e., positional cues). This would have biased spatial learning to what would normally be a female-like strategy. The use of a male-like strategy, which depends on the distant cues outside the maze, would have been prevented by the tall non-transparent walls surrounding the maze. Therefore, under these task conditions, any effects of the digit ratio that was correlated with to male spatial strategy would have been muted or reversed.

In the present study, we examined the relationship between digit ratio and spatial navigation ability by controlling the exact nature of navigation cues in a virtual environment. We propose that a lower digit ratio (more masculine) should be associated with male-like spatial strategies and hence predict superior performance in the presence of directional, but not positional, cues. We hypothesized that the ‘sense of direction’ in spatial navigation is most sensitive to directional cues. We therefore examined the relationship between digit ratio and navigation orientation accuracy in a virtual navigation study with controlled cue types in the environment, containing either all directional cues or all positional cues. In addition, we also measured mental rotation test scores to determine if digit ratio relates to spatial visualization tasks such as the MRT the same way as 3D virtual navigation. Because sex differences in MRT are so well established, we also used the MRT to confirm that a typical cognitive sex difference pattern (i.e., male advantage) could be demonstrated in our sample.

## Materials and Methods

### Ethics Statement

All protocols were approved by the University of California at Berkeley's Committee for the Protection of Human Subjects. All participants gave informed written consent prior to the experiment.

### Participants

Eighty-two undergraduate students (41 females, ages 19.8±1.9; 41 males, ages 19.2±1.1) participated in the virtual navigation task, completed the mental rotation test and had their finger lengths measured. Mental rotation test scores were missing in four males due to computer error. Due to a technical error with the flatbed scanner, the wrong size of the hand images was saved for a subset of subjects. As a result, 3 females and 11 males did not have measurements of their absolute finger lengths. The accuracy of the 2D:4D ratio of these subjects, however, was not affected by the scanner image size. Therefore we included 2D:4D data from all 82 subjects in our analysis. Navigation accuracy data (distance from the hidden target at the end of probe trial) in these participants was previously described in Chai and Jacobs (2009) [46]. Data presented here have not been reported elsewhere.

### Apparatus

We constructed computerized three-dimensional virtual environments (VE) using a commercially available video game engine (Unreal Engine 2 by Epic Games, Raleigh, NC). These environments were presented on a 21-inch computer monitor with participants sitting approximately 55 cm in front of the monitor. Horizontal field of view was approximately 39 degrees and vertical field of view was approximately 30 degrees. Participants used a joystick (Cyborg Evo by Saitek, Bristol, UK) with forward, backward, left-turn and right-turn options to move in the environment. Coordinates of the movement were recorded into a log file every 0.2 s.

### Virtual environments (VE)

**Figure 1:**
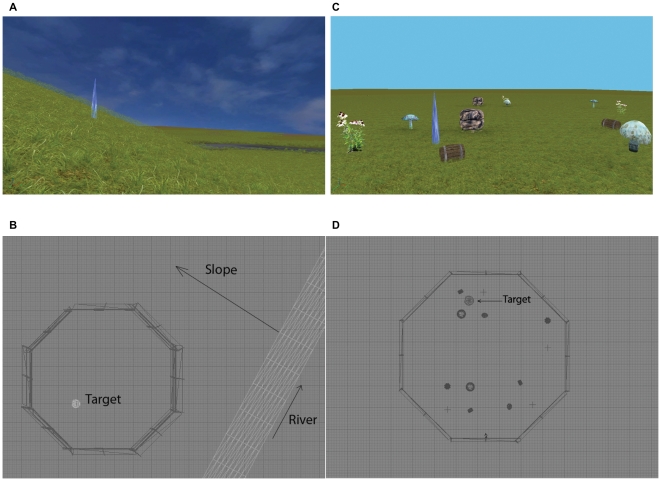
Representative virtual environments for the different cue class trials. Screenshot of a directional-cue trial (A), screenshot of a positional-cue trial (B), schematic of the directional-cue environment (C) (the arrow points up the slope), schematic of a positional-cue environment (D). The blue crystal (target) was located in one of the cue clusters.

The VE task was modeled after the logic of the Morris water maze task, in which the participant must locate a single hidden target in a circular arena. As shown in Figure 1, the VE was a large grassy terrain that contained a test arena surrounded by an octagonal invisible fence that was 18.3 virtual meters in radius. The fence was invisible to ensure an unblocked view of the surrounding cues. The target was a blue spike-like crystal. Two types of environments were constructed, one for the all-directional-cue condition and one for the all-positional-cue condition. In the all-directional-cue environment, the test arena was located on a small hill with a terrain slant of approximately 30 degrees. Other directional cues included a river running at the bottom of the slope, the sun, and a cloud-filled sky (Figure 1A–B). In the all-positional-cue environments, the test arena was situated on a flat terrain with objects such as rocks, small plants, wooden barrels and mushrooms forming object clusters of different configurations within the arena. The target was located in one of the clusters. Because duplicates of the same objects were found at different locations, the task could not be solved by simply associating the target location with a single object (Figure 1C–D).

### Procedure

#### Virtual navigation task

Prior to starting the navigation trials, participants were given a short practice session to familiarize themselves with the VE interface and to practice moving with the joystick. All participants reported they were comfortable with moving in the VE at the end of the practice trials.

Navigation trials commenced immediately after practice trials. There were six trials for each of the two conditions (directional cue and positional cue). The trials were presented in pseudo-random order. Each trial consisted of two phases: a training phase and a probe phase, each 25 s in duration. In the training phase, the target was visible throughout the trial. Participants were told to explore the area and try to memorize the location of the target. Each training phase was followed immediately by the probe phase, in which the target was hidden. Participants had 20 s to approach as closely as possible to the target location in the probe phase. The starting point of the participant was different in the training phase and the probe phase. A timer was displayed on the top left corner of the screen to help participants keep track of time. At the end of the 20 s, the target re-appeared for 5 s to give participants feedback on their performance. If the participant finished the search before 20 s, they were told to remain at their last search location and wait for the feedback at the end of the trial. There was a 10 s inter-trial fixation on a centered cross on the monitor. The location of the target was different in each of the six trials for both directional-cue and positional-cue trials. Each positional-cue trial used different object cues and different object locations.

Orientation ability in virtual navigation was accessed by accuracy in the initial search direction. Heading error, which measures orientation error towards the target, has been used as the classic measure for orientation accuracy in prior navigation studies [47], [48], [49], [50]. Heading error was defined as follows: the deviation of the heading direction at any given point along the path from the optimal direction, ranging from zero (same as the optimal direction) to 180 degrees (opposite from the optimal direction), with chance level at 90 degrees. The average initial heading error from each point along the first 200 virtual units traveled was used in the analysis. The lower the initial heading error, the more accurate the initial orientation.

#### Mental rotation test (MRT)

After the virtual navigation task, participants completed the mental rotation test. We used the Peters redrawn version of the mental rotation test originally constructed by Vandenberg and Kuse (1978) [51]. The object images from the original written test were scanned into jpeg files and displayed on a computer screen. Each problem consisted of one original object and four possible choices, two of which were rotated versions of the original image. Participants were given 3 min to pick the rotated images for 24 problems. One point was given if both correct images were picked as suggested by Peters (1995). This scoring procedure is different from the conventional scoring system by Vandenberg and Kuse (1978).

#### Digit ratio measurements

After the completion of the virtual navigation task and the mental rotation task, digital images of the right hand were obtained for digit length measurements using a flat-bed scanner. Participants were told to place their right hand in the center of the scanner with their palm facing downward and rings removed. The cover of the scanner was then closed before the image of the hand was obtained. No extra pressure was applied. The second (index) and fourth (ring) finger lengths were later measured from the images from the bottom crease where the finger meets the palm to the tip of the finger using the computer software ImageJ 1.37v [52]. The hand images were measured by one of the authors (X.J.C), and by an independent observer. Inter-rater reliability of the digit ratio as measured by intra-class correlation was 0.983. The average of digit ratios from the two raters was used in the data analysis.

## Results

**Figure 2:**
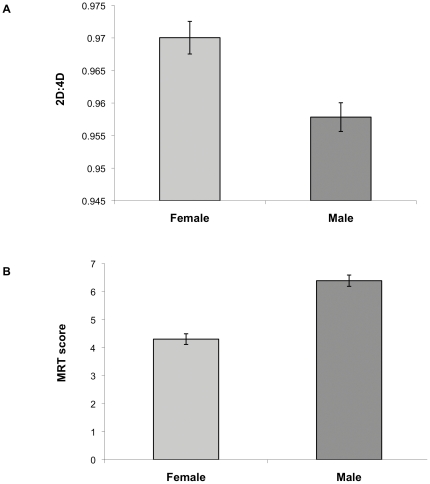
Sex differences in digit ratio (A) and mental rotation score (B). Error bars represent SE.

**Figure 3:**
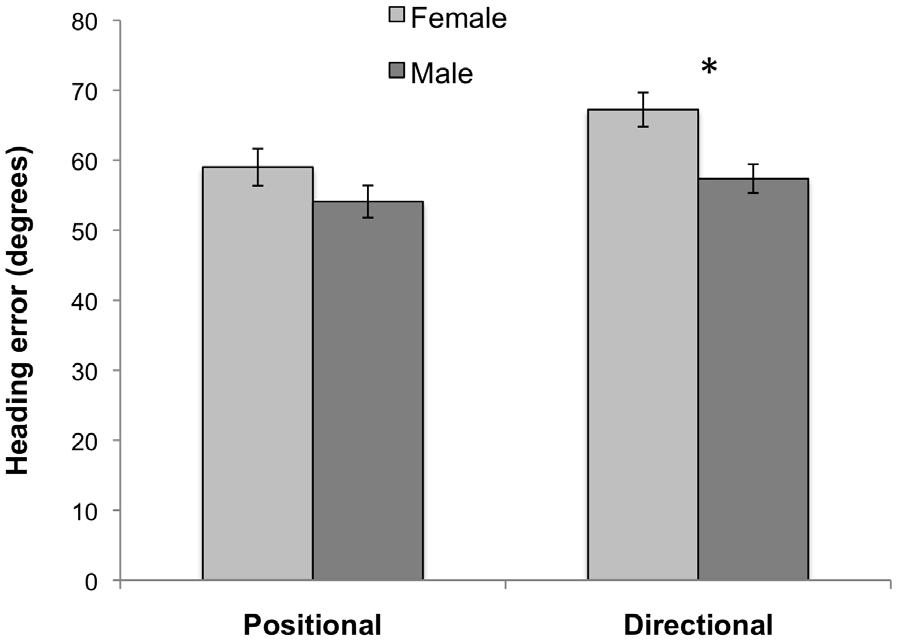
Heading error in all-directional and all-positional cue environments. Error bars represent SE.

Digit ratio was lower in males than females (males, .953±.031; females, .970±.035; *t_80_* = 2.45, *P* = .017; Figure 2A). Males had higher MRT scores than did females (males, 6.59±2.17; females, 4.37±2.41; *t_76_* = 4.27, *P*<.001; Figure 2B). The effect size (Cohen's d) was .51 for the sex difference in 2D:4D and .97 for the sex difference in MRT. In the virtual navigation task, males had lower error in initial heading error (higher accuracy in search direction) compared to females in the directional cue condition (*t_80_* = 3.11, *P* = .003), but not in the positional cue condition (*t_80_* = 1.40, *P* = .17) (Figure 3). MRT scores did not correlate with initial heading error in either positional or directional cue in either males or females (ps>.15).

To assess how digit ratio relates to orientation accuracy in navigation, we conducted an analysis of covariance (ANCOVA) for each cue condition, with heading error as the dependent variable, 2D:4D, sex and the interaction between 2D:4D and sex as independent variables. Within-sex posthoc tests were performed only when there was a significant relationship between 2D:4D and the dependent variable. We also conducted the same analysis for MRT scores. Out of these three tests, the effect of digit ratio was significant for heading error in the directional cue condition (*F_1,77_* = 7.54; *P* = .008; *P* = 0.024 after Bonferroni correction), but not for heading error in the positional cue condition (*F_1,77_* = 1.21; *P* = .28). The relationship between MRT and digit ratio was not significant (*F_1,73_* = 0.63; *P* = .43).

**Figure 4:**
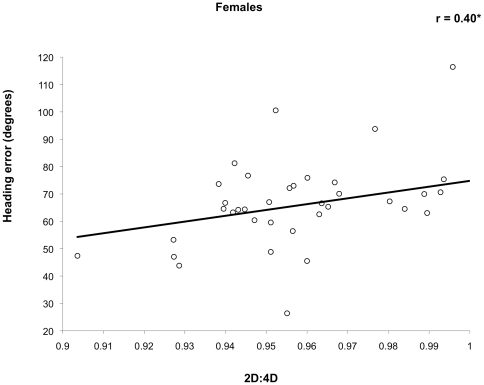
Correlation of digit ratio with virtual navigation initial heading error in females.

We then conducted posthoc correlation tests within each sex only if the primary test describe above was significant, i.e., between digit ratio and with heading error in the directional cue condition. Higher digit ratio was associated with greater error in initial heading in females (r = .40, *P* = .01; Figure 4) but not in males (r = .17, *P* = .29). Females with low digit ratio were therefore more accurate in their initial orientation.

## Discussion

The relationship between digit ratio, a putative marker of organizational hormone effects, and spatial abilities has been controversial. Here we tested participants in two distinct navigational environments (directional or positional cues only), which allowed us to examine this question with greater precision. Our results demonstrate a link between digit ratio and spatial orientation ability in a virtual landscape. In females, digit ratio predicted initial search direction accuracy, i.e., the ‘sense of direction’, when only directional cues were available. This suggests that females with lower digit ratio had better orientation abilities under specific conditions that normally favor males, i.e., when they were required to rely solely on directional cues in the navigational environment. These results are consistent with our hypothesis derived from the parallel map model, which predicts a male advantage in environments with only directional cues. Since directional cues are better encoded and used by males [42], [46], a more masculine digit ratio (i.e., lower) should predict better spatial performance under directional cues. Our results suggest that directional-cue based mapping, the most primitive feature of the cognitive map [44], is organized at an early stage in brain development. Females with lower 2D:4D (putatively higher prenatal androgen levels) may have developed a masculinized cognitive mapping strategy, relying much more on orientation to directional cues than females with higher digit ratios. We did not find evidence for a relationship between male 2D:4D and spatial orientation accuracy. One interpretation for the lack of 2D:4D effect in males is that any “above-threshold” prenatal androgen exposure in males was not beneficial for their spatial ability. Our findings are consistent with data from congenital adrenal hyperplasia (CAH), a condition with high fetal testosterone. CAH males have been shown to have similar or worse spatial performance scores compared to controls, whereas females with CAH showed better spatial ability than unaffected females, and performed at similar levels to unaffected males [20], [27], [53], [54]. CAH females appear to have masculinized 2D:4D, as well as superior spatial ability. It would be interesting to study them in separate cue conditions (directional or positional cue), to test if they have male-like strategy in spatial cue use. Our results are also in accordance with research in rats, where testosterone treatment in neonatal rats improved spatial ability in females but not in males [55]. Neonatal testosterone treatment in females was thought to induce the development of a male-like hippocampus [56].

We did not find correlations between digit ratio and orientation accuracy in the positional cue condition. This negative result was not unexpected. Although some studies [57], [58], [59] have replicated the female advantage in object location memory originally reported by Silverman and Eals (1992) [31], others have failed to reproduce this result [32], [42], [60], [61], [62]. Saucier et al. (2007) suggested the female advantage in object location memory was dependent on whether the objects were close (peri-personal space) or relatively far (extra-personal) from the participant's body [43]. Our previous data suggested that the female advantage in positional cue conditions was less robust than the male advantage in directional cue conditions [42]. Furthermore, we surveyed spatial strategy preferences in the participants in our previous report [46]. Men reported greater preference on a survey (global) representation, which depends heavily on directional cues, whereas preferences on landmark-centered strategies, which depend on positional cues, did not differ between the genders. This lack of sex differences in positional-cue-centered strategy is reflected in the similar accuracy in initial search directions in males and females under the positional cue condition. Our results underscore the importance of defining the types of cues in the environment and the nature of the task, prior to measuring hormonal effects, whether organizational or activational, on sex-specific spatial abilities. Both the Csatho et al. (2003) [29] and Nowak et al. (2010) [26] studies included positional cues in their environment, which could have masked the effect from directional cues. Therefore it was not surprising that they did not find a relationship between digit ratio and spatial ability in the expected direction.

The positional cue condition required greater memory load compared to the directional cue condition. Although this was not optimal, the task was designed this way due to the following reasons: 1) our pilot data suggested that performance was much worse in the directional cue condition than the positional cue condition. Keeping the directional cue environment the same across the experiment was partly an attempt to match the two conditions in level of difficulty; 2) in real life, we typically use the same set of directional cues throughout the course of navigation, whereas when a navigator moves through a spatial environment, they need to update the set of positional cues that guides their navigation.

The mental rotation task has become the standard spatial visualization task for studying the effects of digit ratio on spatial cognition. In concordance with the meta-analysis by Puts et al. (2008) [25], we did not find a correlation between mental rotation scores and digit ratio. Our findings suggest that sub-components of spatial ability should be examined separately in future studies. We propose that the literature has relied on a low resolution definition of spatial ability, conflating behaviors such as spatial orientation with mental rotation. The contribution of the parallel map model is to distinguish finer subcategories of spatial orientation, i.e., directional bearing, which in this model is a trait more accurately encoded and performed by males. We suggest that using finer grained cognitive tests is the way forward to resolving the inconsistent pattern of results in the literature.

Activational effects of adult circulation hormones have been reported to affect spatial ability [62], [63]. Although without directly measuring circulating gonadal hormone levels, we can not completely rule out the alternative interpretation that female participants with low 2D:4D in our study had lower circulating estrogen or high testosterone, other evidence suggest this is unlikely to be the case. A recent study that included a large data sample (160 women and 177 men) did not find a correlation between salivary testosterone and spatial ability [64]. Moreover, a meta-analysis by Honekopp et al. (2007) [5] found no association between adult sex hormone and digit ratio. Therefore the correlation between digit ratio and spatial ability observed in the present study is more likely to reflect the organization effect of testosterone on spatial cognition. However, it is important to point out that the mechanism linking digit ratio and sexually dimorphic traits is still under debate. Although there is evidence that suggests androgen receptor gene may influence digit ratio [6], [65], several studies have not replicated this finding and suggest other mechanisms may be involved [7], [66], [67]. Neonatal testosterone levels may also modulate 2D:4D [68]. Activational effect of testosterone on social cognition has been shown to be dependent on 2D:4D, possibly being facilitated by the early organizational effect of testosterone [69]. Future studies are needed to elucidate the exact nature of the relationship between 2D:4D and the organizational and activational effects of sex hormone on cognition.
